# Use of Common Inflammatory Markers in the Long-Term Screening of Total Hip Arthroprosthesis Infections: Our Experience

**DOI:** 10.1155/2017/9679470

**Published:** 2017-08-23

**Authors:** Gabriele Falzarano, Antonio Piscopo, Predrag Grubor, Giuseppe Rollo, Antonio Medici, Valerio Pipola, Michele Bisaccia, Auro Caraffa, Elizabeth Mary Barron, Francesco Nobile, Raffaele Cioffi, Luigi Meccariello

**Affiliations:** ^1^Department of Orthopedics and Traumatology, Azienda Ospedaliera “Gaetano Rummo”, Benevento, Italy; ^2^Department of Orthopedics and Traumatology, Sacro Cuore di Gesù Fatebenefratelli Hospital, Benevento, Italy; ^3^Clinic of Traumatology, University Hospital Clinical Center Banja Luka, Banja Luka, Bosnia and Herzegovina; ^4^Department of Orthopedics and Traumatology, Vito Fazzi Hospital, Lecce, Italy; ^5^Rizzoli Orthopedic Institute, University of Bologna, Bologna, Italy; ^6^Division of Orthopedics and Trauma Surgery, University of Perugia, S. Maria della Misericordia Hospital, Perugia, Italy; ^7^School of Medicine, Central Michigan University, Mount Pleasant, MI, USA; ^8^Department of Orthopedics and Traumatology, Hospital “Santa Maria alla Gruccia”, Montevarchi, Arezzo, Italy

## Abstract

Orthopedic implants have become essential components of modern medicine. The risk of infection of total hip arthroplasty (THA) is 1.5%−2%. Are the C-reactive protein (CRP), the erythrocyte sedimentation rate (ESR), and procalcitonin (PCT) good markers for THA infection screenings? From February 2009 to December 2012 at our Department of Orthopedics and Traumatology, 1248 patients were treated with THA. No prosthesis was cemented. All patients received antibiotic prophylaxis. All patients were discharged approximately 7.4 days after surgery with this clinical and radiographic follow-up program at 15 days and 1, 3, 6, 12, 24, and 36 months after surgery. Blood samples to determine ESR, CRP, and PCT values were taken at 1 hour before surgery and 15 days and 1, 3, 6, 12, 24, and 36 months after surgery. During follow-ups there were 22 cases of THA infections; according the Widmer classification, infections are hematogenous ones in 16 cases, late chronic ones in 5 cases, and early postoperative ones in 1 case. In all cases the three markers were considered positive; in 6 cases there were no radiological signs of septic loosening. ESR, CRP, and PCT proved to have a greater diagnostic accuracy than X-rays in predicting late chronic and early postoperative infections. These markers are valuable support for the surgeon in monitoring the prosthetic implant lifespan.

## 1. Introduction

The number of primary total hip arthroplasties (THAs) performed in the United States each year continues to increase, as does the incidence of septic complications. The changing profile of antibiotic resistant bacteria has made the prevention and the treatment of primary THA infections increasingly complex [1]. The incidence of PJI (prosthetic joint infections) varies depending on the joint involved; the rate of arthroplasties becoming infected is as follows: 1.7% of primary and 3.2% of nonprimary hip arthroplasties [2]. A correct and early diagnosis is essential in order to provide the most appropriate therapy. If a correct and timely microbiological diagnosis of infections is done within 4 weeks, it could be possible to follow a conservative approach on the prosthesis, since microorganisms are not yet organized in biofilms. A delayed diagnosis (>4 weeks) of early and late infections involves the necessity of prosthesis removal [3] due to the production of a structurated and mature microbial biofilm. Biofilm is an aggregate of microcolony of microbial cells adherent to a living or nonliving surface embedded in an extracellular polymeric matrix. Biofilm renders bacteria highly tolerant to antibiotics and host defenses [4].

The definition of PJI was recently revised by the International Consensus Group on Periprosthetic Joint Infection. According to PJI Consensus Group, patients should be considered to have a PJI if they meet one of the major criteria or at least three of the minor criteria. Major criteria are as follows: two positive periprosthetic cultures with phenotypically identical organisms; a sinus tract communicating with the joint. Minor criteria are as follows: elevated serum C-reactive protein and erythrocyte sedimentation rate; elevated synovial fluid white blood cell (WBC) count or ++ change on leukocyte esterase test strip; elevated synovial fluid polymorphonuclear neutrophil percentage; positive histological analysis of periprosthetic tissue; a single positive culture [5].

The aim of our study is to investigate the rationale and the utility of a long-term screening of prosthetic hip infections by use of common inflammatory markers [3]: erythrocyte sedimentation rate (ESR), C-reactive protein (CRP), and procalcitonin (PCT).

## 2. Material and Methods

From January 2009 to December 2012 a cohort of 1248 patients, who had undergone single hip arthroplasty at Department of Orthopedics and Traumatology of the “G. Rummo” Hospital of Benevento, the Sacred Heart Hospital Fatebenefratelli (Benevento, Italy), and the Clinical Center of Banja Luka (BIH), had been prospectively followed to detect the onset of prosthesis infection.

The population of our group at the time of the implant (THA) had an average age of 73.4 years (range 21–85); the relationship between sexes (M : F) was of 1.15 (658 : 570). The relationship between osteoarthritis and femoral fractures was 2.59 (900 : 348). The ASA physical status classification system was used to assess the anesthesiological risk of the patients (ASA I: 338 (27.08%); ASA II: 792 (63.46%); and ASA III: 118 (9.46%)) and the number of patients transferred to the intensive care unit after surgery, 59 (4.73%), with an average stay of 2.8 days (range 1–6 days). The average days of hospital stay were 6.7 days (range 4–15 days). 40 patients (3.2%) were operated on with anterior surgical access, 926 (74.2%) with direct lateral surgical approach, and 282 (22.6%) with posterior-lateral approach. On average, the surgical wound was 11.6 cm long (range of 10.3–16.5 cm). The average time for the THA surgery was 56.3 minutes (range 48.3–90.8 minutes). 523 patients (41.19%) received general anesthesia and 725 patients (58.81%) spinal anesthesia (Table 1).

No THA was cemented. All patients received short term cefazolin for antibiotic prophylaxis (2 gr before the surgery and 1 gr every 8 hours for five times after the surgery). The THA surfaces were the following: ceramic on polyethylene in 53.7% (*n* = 670 patients), ceramic on ceramic in 38.6% (*n* = 482 patients), and metal on metal in 7.7% (*n* = 96 patients).

Patients were treated according to the ethical standards of the Helsinki Declaration and were invited to read, understand, and sign the informed consent form.

The number of comorbidities was 1561 (Table 2). Cardiovascular diseases which affected 356 patients (28.53%) were the most frequent comorbidities (Table 2).

Follow-ups were performed with clinical and radiographic evaluations with pelvic and hip projections (AP, LL, and axial femur) at 15 days, 1 month, 3 months, 6 months, 12 months, 24 months, and 36 months after surgery. Blood tests were conducted to determine ESR, CRP, and PCT values: one hour before surgery, 15 days, 1 month, 3 months, 6 months, 12 months, 24 months, and 36 months after surgery. During this period, complications were evaluated and the possible infections were listed according to the Widmer classification.

The evaluation endpoint was set at 36 months. The exclusion criteria were the voluntary withdrawal from the scheduled follow-up program.

Furthermore, to confirm suspected infections, the surgeon examines the joint fluid (cytochemical and microbiological), the histological and microbiological periprosthetic tissue, and the prosthetic components.

No PJI Consensus Group criteria were followed in the suspicion of PJI because the study started before their publication and the study protocol was set as explained above.

All parameters were recorded into a spreadsheet for further processing and statistical analysis.

Descriptive statistics were used to summarize the characteristics of the study group and subgroups, including means and standard deviations of all continuous variables. The *t*-test was used to compare continuous outcomes. The Chi-square test or Fisher (in subgroups smaller than 10 patients) exact test was used to compare categorical variables.

The statistical significance was defined as *p* < 0.05.

## 3. Results

**Figure 1:**
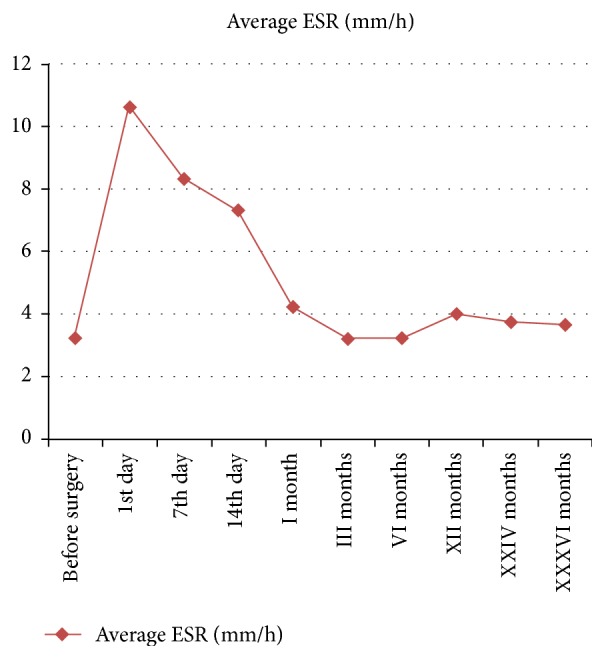
Trend of ESR during 36 months of follow-up.

**Figure 2:**
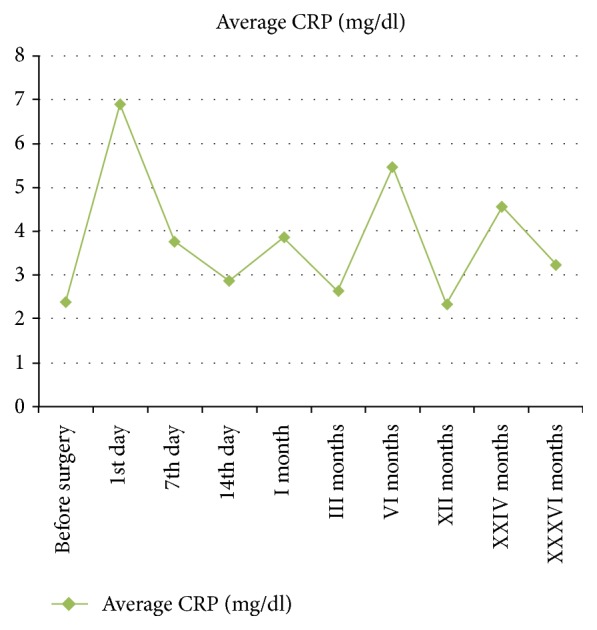
Trend of CRP during 36 months of follow-up.

**Figure 3:**
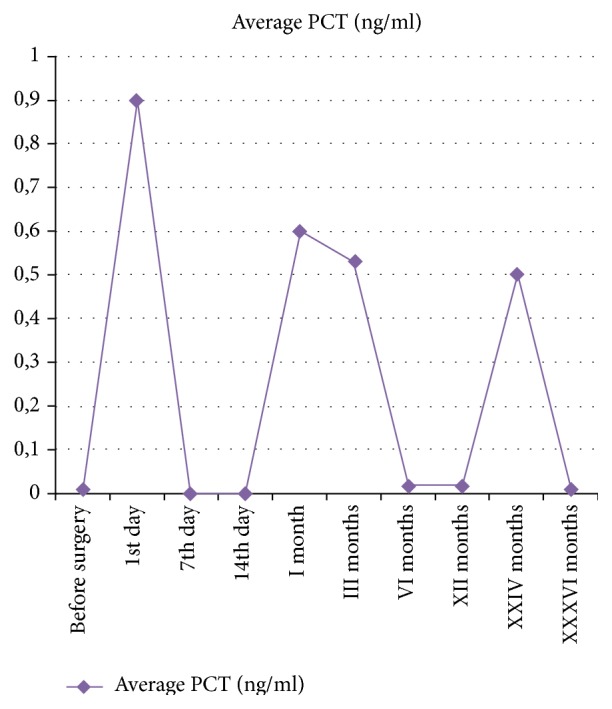
Trend of PCT during 36 months of follow-up.

There were 387 complications not related to the THA system (Table 3). The most common complication during the 36-month period was the infection of the urinary tract in 168 patients (13.46%). 4 of them died during follow-up (Table 3). The ESR, CRP, and procalcitonin trends, shown in Figures 1, 2, and 3, are on average not dissimilar to the limits of the work. 1.76% of the patients (*n* = 22) had a PTA infection.

According to the Widmer classification (Table 4), there were one case of implant infection in the first four weeks after surgery (with WBCs: 24,000; ESR: 36; CRP: 35; PCT: 2.3), 5 cases of prosthetic infection between 5 weeks and the 23 months after surgery (average values: white blood cells: 18,356; ESR: 52.6; CRP: 42.3; PCT: 2.8), and 16 cases of prosthetic infection after 24 months after surgery (average values: white blood cells: 16,300; ESR: 56; CRP: 22.9; 14.2 PCT).

We had 20 patients with false positive values of CRP: 4 during the first month; 14 between the first month and 2 years; 2 patients after two years.

Before the prosthetic implant revision, aspiration under fluoroscopy of the intra-articular hip fluid was performed to determine the number of white blood cells and the presence of pathogens. The pathogens were classified as follows: coagulase-negative staphylococci in 12 cases (54.54%), Gram-negative bacilli in 7 cases (31.82%), and enterococci in 3 patients (13.64%). After 5 days from the intra-articular aspiration, an implant revision was performed in two stages for late chronic and hematogenous infections and 1 stage for the early infection. There were no new cases of reinfection after revision. We did not perform intra-articular aspiration to all patients but only in those whose value of white blood cell was over 10.000 or those with elevated value of CRP and ESR. In some patients we performed an intra-articular aspiration without finding a joint infection because of false positive values of ESR and CRP due to other conditions that could rise their value [6].

## 4. Discussion 

The total hip arthroplasty infections represent a serious problem given the increasing number of implants performed each year for both arthritis and hip fractures. Early detection of infection is the main objective orthopedists need in order to adopt an appropriate treatment procedure.

The incidence of primary hip arthroplasty infections of this study is 1.76%. This value is in line with the incidence of hip prosthetic infections present in the literature which is comprised between 0.3% and 1.7% of primary implants [3, 7]. Patients developing an infection at follow-up had a history of more than three comorbidities [8–12].

Prosthetic infections are usually listed according to the Widmer classification: early postoperative if they occur during the first 2–4 weeks after surgery; late chronic after the first month; hematogenous after two years [1].

The purpose of the study is to evaluate the hematological parameters showing an early diagnosis of primary implant suspected infection. The following indexes are used: CRP, ESR, and PCT. CRP and PCT show a statistically significant peak during the first week after primary hip arthroplasty and then return to similar preintervention values in the first 14 days [3]. CRP has no sensitivity of 100%, so some cases of infection may go unrecognized because of low-grade infections or due to encapsulated bacteria associated with a less intense systemic response with a more modest rise of inflammatory markers. The PCT also increases the sensitivity of CRP and permits avoiding false positive results, as it is a more specific marker for bacterial infections [5], although the trauma and surgery show a transient increase. The assessment of the time of the laboratory indexes permits pointing out, in cases of infection, that the average CRP, ESR, and PCT values show a statistically significant peak compared to the average of uninfected patients' values at the same time. The early postoperative infections resulted in an increase of CRP, ESR, and PCT greater than that associated with trauma and surgery [13] which occurs in the first 2 weeks after surgery. This is an extremely important fact because it emphasizes the importance of CRP, ESR, and PCT for the diagnosis of early infections by showing suspicious markers which enable the orthopedic surgeon to implement the best diagnostic-therapeutic protocol.

In a study conducted at Isfahan's educational treatment centers from 2009 to 2011, 80 patients, candidates for THA and TKA, were examined. 35 patients were candidates for TKA and 45 others for THA. ESR and CRP were analyzed on the day before surgery, the day of operation, and after 1, 2, 5, and 15 days and 1, 3, 6, and 12 months after total joint replacement. The mean ESR had an upward trend during the first 5 days and then decreased gradually, lasting up to 3 months. After 1 year it increased to a level higher than that before operation but its value was not statistically significant. The same happened for CRP. Patients with high ESR and CRP that do not follow the normalization process have to be studied to evaluate the presence of prosthetic infection [14].

In a study done in an academic center in Spain on patients who had undergone THA and TKA operations, the level of CRP was measured before operation and 1, 2, 3, 15, 42, and 150 days after operation, in which the maximum level was related to 2 days after surgery. CRP returned to preoperative level in 150 days. Patients who showed an increase of CRP after 3 days with a persisting level after the 42nd day were worthy of study to evaluate the presence of prosthetic infection [15].

The use of these markers in the screening of THA infections is associated with a percentage of false positive and false negative results. False positives are linked to the increasing of these markers both in inflammatory and in infection processes. Instead, false negatives depend on the fact that in same patients they rise only in a clinical infection. False positive and false negative results depend on the sensibility and specificity of ESR, CRP, and PCT. ESR has a sensitivity of 0.93 and a specificity of 0.86; CRP has a sensitivity of 0.91 and a specificity of 0.86 [16].

In our retrospective cohort study, discrepancies between C-reactive protein and erythrocyte sedimentation rate have been reported in 12.5% of patients. Patients with raised C-reactive protein and a normal erythrocyte sedimentation rate usually have infection but some have other tissue damage (e.g., myocardial infarction or venous thromboembolism). These discrepancies may be due to timing, with the rise in C-reactive protein manifesting itself before the sedimentation rate elevates, or simply because the sedimentation rate does not change with minor inflammation. Patients with a high erythrocyte sedimentation rate and normal C-reactive protein mostly have conditions without demonstrable systemic inflammation such as malignancy [17].

However, there are two circumstances when the sedimentation rate can be a better marker of an inflammatory process: some low-grade bone and joint infections (e.g., in joint prosthesis infections due to low-level pathogens such as coagulase-negative staphylococci); autoimmune disease, in particular some people with systemic lupus erythematosus [6].

Seriate controls of such markers in the first 4 weeks after surgery fairly indicate a primary implant suspected infection as the study of the temporal trend may help in discriminating between infection and postoperative inflammatory phase [14]. In addition, the use of these markers makes it possible to avoid the indiscriminate use of imaging tests such XR controls, scintigraphy [15], and PET/CT and resort to them only when there is reasonable suspect of infection [7].

Intra-articular aspiration can give false positive results. In fact, Meermans and Haddad performed a prospective study of 120 patients who underwent aspiration and biopsy for suspected joint infection (64 with THAs and 56 with TKAs). The sensitivity reported was 83% for aspiration, 79% for biopsy, and 90% for the combination of both techniques [18].

In a prospective study conducted at the ENDO Klinik, Bonanzinga et al. enrolled 156 patients with chronically (>90 days) painful total joint arthroplasties (65 TKAs, 91 THAs). Diagnosis of PJI was made according to the PJI Consensus Group criteria. Patients diagnosed as having PJI underwent a single-stage direct exchange following the ENDO Klinik protocol if the microorganisms were known in advance or two-stage surgery if the bacteriology was not known after preoperative diagnostics. Intraoperative aspiration was performed after surgical incision, preparation of soft tissues, and exposure of capsule without opening the joint and sending to laboratory for the alpha-defensin immunoassay test. In 29 patients with PJI confirmed intraoperatively, alpha-defensin assay was positive in 28 patients and negative in 1 patient. On the other hand, in 127 confirmed aseptic painful total joint arthroplasties alpha-defensin test was positive in four cases (2 with metallosis, 1 with severe polyethylene wear with osteolysis, and 1 with unknown origin). Statistical analysis reveals that alpha-defensin immune assay sensibility was 97%, specificity was 97%, positive predictive value was 88%, and the negative predictive value was 99% [19].

In a retrospective study including data from 106 hip and knee arthroplasties with PJI diagnosed more than 90 days after primary surgery, alpha-defensin assay showed higher sensitivity in diagnosing PJI among patients given antibiotics when compared with ESR, CRP, fluid PMN%, and fluid culture [20].

Unlike the cases of late chronic and hematogenous infections that occur from the first month after surgery in which the use of laboratory indexes, object of study, is definitely of help in confirming the suspected diagnosis, in these cases, given the striking clinical manifestations, it would be more beneficial to use imaging tests such as X-rays, scans, and eventually PET/CT showing signs of implant infection rather than resorting to seriated CRP, ESR, and PCT [7, 16, 17] controls. The analysis of such markers therefore has a favorable impact on cost management for patients with early infections, rather than using imaging methods such X-rays, scans, and PET/CT. For patients with late chronic and hematogenous infections undergoing a long follow-up through regular CRP, ESR, and PCT evaluations and for those undergoing primary hip arthroplasty, the diagnostic suspect may be helpful but it represents an unnecessary expense if compared to the costs of diagnostic imaging methods [7, 21]. Synovial fluid biomarkers, such as alpha-defensin, represent a breakthrough in the scenario of late chronic and hematogenous PJI and may represent in the next future a valid tool in the diagnosis of PJI.

In our experience, only in the cases of late chronic infections we had X-ray findings of septic loosening. In the other cases, X-rays were normal.

In conclusion, according to our experience, we suggest the use of seriated CRP, ESR, and PCT controls for patients undergoing total hip arthroplasty in the first 4 weeks after surgery; their evaluation during this period allows discriminating between early postoperative infection and reactive inflammatory phenomena and implementing, after an initial assessment of these indexes, appropriate investigations with diagnostic imaging if there is a suspect of late chronic and hematogenous infection.

## Figures and Tables

**Table 1:** Description of population.

Description	
Numbers of patients	1248
Average age of patients	73,40 years
Range of age	21–85 years
Gender ratio (M : F)	1,15 (658 : 570)
Diseases ratio (arthritis/fractures)	2,59 (900 : 348)
	ASA I: 338 (27,08%)
ASA physical status classification system	ASA II: 792 (63,46%)
	ASA III: 118 (9,46%)
Number of patients who needed intensive care after surgery	59 (4,73%)
Average days in intensive care	2,8
Range of days in intensive care	1–6
Average days of hospitalization	6,7
Range of days of hospitalization	4–15
	Anterior approach: 40 (3,20%)
Surgical approach	Laterolateral approach: 926 (74,20%)
	Posterolateral approach: 282 (22,6%)
Average length of surgical wound	11.6 cm
Range of length of surgical wound	10.3–16.5 cm
Average length of surgery	56.3 minutes
Range of length of surgery	48.3–90.8 minutes
General anesthesia	523 (41,19%)
Spinal anesthesia	725 (58,81%)

**Table 2:** Number of comorbidities (%).

Respiratory disease	256 (20,51%)
Renal disease	108 (8,65%)
Diabetes mellitus	72 (5,77%)
Rheumatoid disease	243 (19,47%)
Parkinson disease	16 (1,28%)
Severe mental deterioration in old age	3 (0,24%)
Paget disease	12 (0,96%)
Current smokers	160 (12,82%)
Enteral steroids	281 (22,51%)

Number of comorbidities in patient	

1	476
2	894
≥3	191

**Table 3:** Number of perioperative complications.

	Number (%)
Chest infection	24 (1,92%)
Cardiac failure	60 (4,80%)
DVT/PE	27 (2,16%)
Urinary tract infection	168 (13,46%)
Gastrointestinal haemorrhage	27 (2,16%)
Myocardial infarction	54 (4,33%)
Stroke	27 (2,16%)
Number of complications	
1	170
2	89
≥3	128
Total of complications	387
Died before the second year of follow-up	4

**Table 4:** Description of THA infection.

Infection category	Typical onset after surgery	Type	Signs and symptoms	Representative microorganism
Early postoperative	≤2–4 weeks	Acute (type I)	Persistent pain after surgery, fever, redness, swelling after surgery	*Staphylococcus aureus*, coagulase-negative staphylococci
Late chronic	≥1 month	Chronic (type II)	Insidious onset, persisting pain after surgery	Coagulase-negative staphylococci, *Propionibacterium* species, anaerobes, *S. aureus*
Hematogenous	>2 years	Acute (type III)	Fever, pain, redness, swelling after a long period of wellness	Streptococci, *S. aureus*, gram-negative bacilli
